# Advancing Adolescent and Young Adult HIV Prevention and Care and Treatment Through Use of Multi-level Theories and Frameworks: A Scoping Review and Adapted HIV Ecological Framework

**DOI:** 10.1007/s10461-023-04255-1

**Published:** 2024-02-14

**Authors:** Julie A. Denison, Kalai Willis, Stephanie M. DeLong, Kirsty M. Sievwright, Allison L. Agwu, Renata Arrington-Sanders, Michelle R. Kaufman, Sandeep Prabhu, Ashlie M. Williams, Errol L. Fields, Kamila A. Alexander, Lana Lee, Cui Yang, The Johns Hopkins University Center for AIDS Research Adolescent and Young Adult Scientific Working Group

**Affiliations:** 1 Department of International Health, Johns Hopkins Bloomberg School of Public Health, 615 N. Wolfe Street. Room E5546, Baltimore, MD 21205 USA; 2 Department of Epidemiology, Johns Hopkins Bloomberg School of Public Health, Baltimore, MD USA; 3 Department of Pediatrics, Johns Hopkins School of Medicine, Baltimore, MD USA; 4 Department of Health, Behavior, and Society, Johns Hopkins Bloomberg School of Public Health, Baltimore, MD USA; 5 Johns Hopkins School of Nursing, Baltimore, MD USA; 6 Adult Clinical Branch, Office of HIV/AIDS, United States Agency for International Development, Washington, DC USA; 7 HIV Center for Clinical and Behavioral Studies, New York State Psychiatric Institute and Columbia University, New York, US

**Keywords:** HIV, Adolescents, Young adults, Multi-level theories, Review

## Abstract

While multi-level theories and frameworks have become a cornerstone in broader efforts to address HIV inequities, little is known regarding their application in adolescent and young adult (AYA) HIV research. To address this gap, we conducted a scoping review to assess the use and application of multi-level theories and frameworks in AYA HIV prevention and care and treatment empirical research. We systematically searched five databases for articles published between 2010 and May 2020, screened abstracts, and reviewed eligible full-text articles for inclusion. Of the 5890 citations identified, 1706 underwent full-text review and 88 met the inclusion criteria: 70 focused on HIV prevention, with only 14 on care and treatment, 2 on both HIV prevention and care and treatment, and 2 on HIV-affected AYA. Most authors described the theory-based multi-level framework as informing their data analysis, with only 12 describing it as informing/guiding an intervention. More than seventy different multi-level theories were described, with 38% utilizing socio-ecological models or the eco-developmental theory. Findings were used to inform the adaptation of an AYA World Health Organization multi-level framework specifically to guide AYA HIV research.

## Introduction

In an era where advances in HIV prevention, care, and treatment have led to calls for the end of the AIDS epidemic [1, 2], the disproportionate burden of HIV on adolescents and young adults (AYA) continues to grow. In 2020, an estimated 410,000 young people aged 10 to 24 years newly acquired HIV worldwide [3], and only slightly more than half of adolescents living with HIV (940,000/1.7 million) received antiretroviral therapy (ART) [4]. In response to these stark statistics, the Johns Hopkins University (JHU) Center for AIDS Research (CFAR) Adolescent and Young Adult Scientific Working Group (AYA SWG) was convened with the mission to promote interdisciplinary research collaborations across the intersecting domains of AYA health and HIV (https://hopkinscfar.org/science-cores/adolescent-young-adult-swg/). Early in the formation of the AYA SWG, members across the JHU schools of medicine, nursing, and public health, shared the different conceptual theories and frameworks they used in their work with young people. This process highlighted a gap with multi-level theories (defined here as theories and/or frameworks encompassing several tiers of influence), needed to guide research and programs for AYA HIV prevention, care, and treatment. The AYA SWG decided to address this gap as presented in this paper.

Multi-level theories, such as Socio-Ecological Models (SEMs), are an important tool for identifying how individuals interact with their environment, and how the interplay of risk and protective factors across levels (e.g., individual, interpersonal, environmental, macrosocial) influences and provides intervention points for health behaviors and outcomes. Several HIV-specific SEMs [5–11] provide a strong rationale and evidence that intervening on multiple levels can mitigate HIV acquisition more than individual-level approaches alone [12, 13]. However, these HIV-specific SEMs are not tailored to AYA and their distinct developmental stages. Furthermore, there are SEMs that focus on children and youth development, including Bronfenbrenner’s, [14]; Blum et al. [15], and the World Health Organization’s (WHO) Ecological Model of the Determinants of Adolescent Health and Development [16], but these broader AYA SEMs do not address HIV explicitly.

We need HIV specific, theory-based multi-level research and programs that address the profound growth that AYA experience. Adolescent development, from early, middle, and late adolescence through young adulthood, is characterized by an expanding ability to think abstractly, plan for the future, and establish a secure identity. Adolescence can also be a time for vulnerability due to an inability to link cause with effects of behavior and to incorporate risk perception into behavior [16]. These changes may influence AYA exposure to HIV risk and protective factors. At the same time, AYA access to services, social roles in different settings, and protections under the law may also be shifting. The extent to which AYA’s health and well-being are fostered or hindered during these years has consequences across the life course, as well as into the life of the next generation [17].

A first step toward achieving an AYA HIV specific multi-level framework is to review if and how researchers in the field of AYA HIV have applied such frameworks in their research; and in doing so, identify potential gaps. To address this need, we conducted a scoping review of the literature from 2010 to May 2020. The objectives of this paper are to present the findings of the scoping review of AYA HIV prevention and care and treatment empirical research that directly state use of named multi-level theories and frameworks, also describing how the theory or framework was used, and how it was applied in those studies assessed. Results of this scoping review were used by the JHU CFAR AYA SWG to adapt an existing AYA multi-level framework to further tailor it to AYA HIV prevention and care and treatment. The hope was that the revised framework could be used as an interdisciplinary tool to guide and generate thought related to AYA HIV prevention and treatment researchers in their analyses, study designs, and interventions. This AYA HIV specific framework is presented in this paper.

## Methods

### Data Source

We searched the following five electronic databases: PubMed, Embase, CINAHL Plus (Ebsco), PsycINFO, and Sociological Abstracts through May 2020. For each database, a search strategy was developed in collaboration with an Informationist at the JHU Welch Medical Library to identify articles that included multi-level approaches in the context of HIV prevention and care and treatment among AYA. MeSH terms, when available, were searched for HIV, adolescent, and theoretical frameworks. Otherwise, searches were restricted to titles and abstracts using the following algorithm: {“HIV” OR “human immunodeficiency virus” OR “AIDS”} AND {“adolescent” OR “youth” OR “young adult” OR “teen” OR “student”} AND {“theoretical model” OR “conceptual model” OR “theoretical framework” OR “conceptual framework” OR “social ecological model” OR “socio ecological model” OR “multi-level” OR “multilevel”}. Truncation was used as appropriate (see Appendix Table 4 for search terms). All search results were imported into an EndNote database prior to coding with duplicate articles deleted. Articles were then uploaded into Covidence [18] for screening and review.

### Inclusion Criteria

**Fig. 1:**
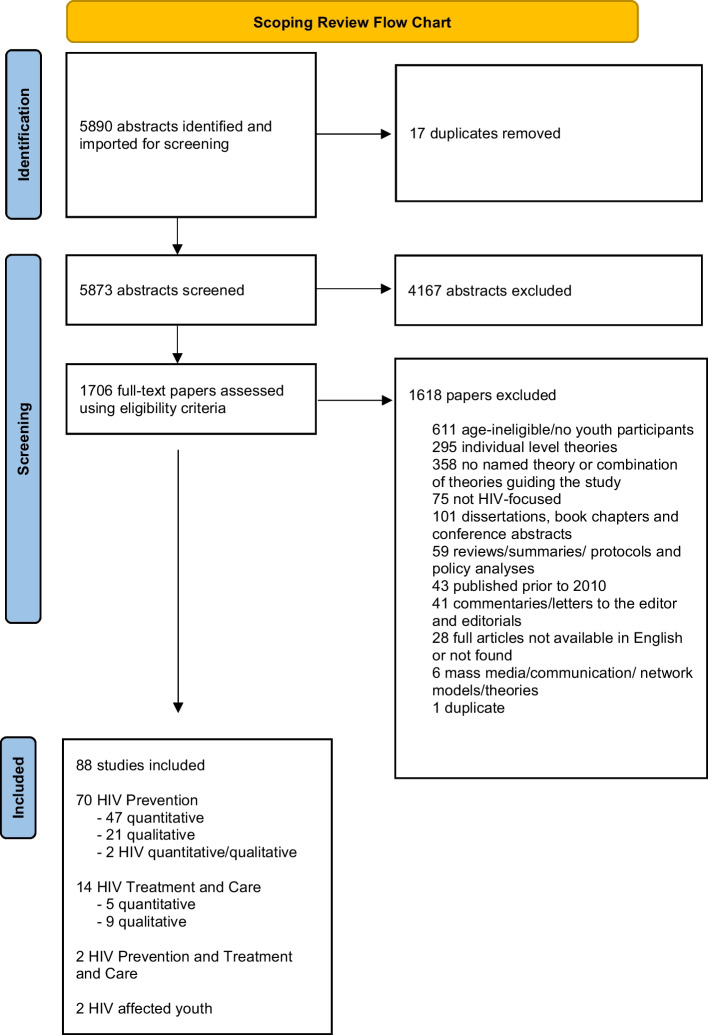
Scoping review prisma flow chart

The review of records for inclusion was sequential. After initial screening in Covidence to exclude articles not related to HIV or with a mean participant age > 25 years, a full text double review was conducted (by authors SD, JAD, KW, CY, KMS) to ensure the remaining articles met the following inclusion criteria: (1) the study population consisted predominantly of AYA aged 10–24 years based on the WHO definition (mean or median age fell between 10 and 24 years or 50% or more of the study population were AYA); (2) were HIV-focused; (3) presented a named multi-level theory (defined here as theories and/or frameworks encompassing several tiers of influence); and (4) were published in 2010–May 2020. Figure 1 details the reasons for exclusions, with most articles excluded based on age of participants, presenting only individual-level theories (e.g., Health Belief Model, Theory of Planned Behavior, Social Cognitive Theory), and not having a named theory or combination of theories that addressed factors on multiple levels. We reviewed articles published from 2010 to May 2020 to account for the following advances: (1) the availability of some prominent AYA—general health SEMs [15, 16]; (2) an increasing focus and awareness of AYA as a critical population to achieve the UNAIDS 90-90-90 HIV goals [19, 20]; and (3) the emerging application of multi-level theories and frameworks to HIV, including advances in technology and the push for combination interventions. Discrepancies between the two reviewers were then resolved by a third reviewer and, if needed, discussion and consensus among all three reviewers.

### Data Extraction

Two independent reviewers extracted data from each article that met the inclusion criteria. Discrepancies were resolved by a third reviewer to check for consistency. Data extracted from each article included author name; year of publication; location(s) of the study; whether the article focused on HIV prevention or care and treatment, or both; the names of the multi-level approach(es) used; and a concise description on how the approaches were used in the article as described by the authors. Data were extracted using a standard extraction form in Excel.

## Results

Of the initial 5890 articles of the search, 1706 underwent full text review, after which 88 met the inclusion criteria. Details of the search and screening results are presented in Figure 1, and details of the included articles are provided in Tables 1, 2, 3. Altogether, 70 of the 88 included articles focused on HIV prevention [21–89

90], 14 on HIV care and treatment [91–104], 2 on both HIV prevention and care and treatment [105, 106] and 2 with HIV affected youth [107, 108]. Out of the 70 HIV prevention-focused articles (Tables 1, 2, 3), 31 were studies conducted in sub-Saharan Africa (SSA), 30 in North America, 5 in South Asia, and 4 in the Caribbean Islands. For the HIV care and treatment-focused articles, 9 were from SSA, 2 from North America, 2 from Asia and 1 from South America. In terms of methods, 54 articles presented quantitative data only, 30 articles presented qualitative data only, and 4 articles presented both quantitative and qualitative data. Nine of the fourteen (64%) articles on care and treatment only presented findings from qualitative research, in comparison to 21 of 70 (30%) of the HIV prevention articles. Additionally, most care and treatment articles (79%) were published in 2017 or later, while a smaller proportion (37%) of the prevention articles were published during those years, with most published prior to 2017. In terms of gender, 52 of the 88 articles enrolled both male and female AYA, 17 studies enrolled females only, 13 studies enrolled males only, and 7 studies included transgender youth (data not shown).

**Table 1:** List of HIV prevention quantitative articles identified (n = 47)

	Year	Location(s)	Name of multi-level theory/framework	How multi-level theory/framework was used
Babalola	2011	Multiple countries in SSA	• Boerma and Weir’s proximate determinants framework	Informed data analysis
Bauermeister	2011	USA	• Social disorganization theory	Informed measure creation and data analysis
Brennan	2012	USA	• Syndemic theory	Informed measures and data analysis
Burton	2019	Canada	• Social ecological model	Informed measure selection and data analysis
Carlson	2012	Tanzania	• Sen’s capability theory	Informed intervention development and analysis
			• Habermas’ communication action theory	
			• Boal’s participatory drama method	
			• Bronfenbrenner’s ecological theory	
			• Bandura’s theory of self and collective efficacy	
Cheruiyot	2019	Kenya	• Andersen and Newman’s framework of healthcare utilization	Informed measures selected and data analysis
Cho	2019	Kenya	• The four bases of gendered power	Informed data analysis
Cluver	2013	South Africa	• Interactive theoretical model developed by the research team	Study tested the interactive theoretical model that was informed by the other named theories/models
			• Sameroff’s transactional theory of impacts of parental psychopathology	
			• Cicchetti’s ecological/transactional model of impacts of child maltreatment	
			• Rutter’s pathways theory to identify direct and indirect chain effects of childhood adversity	
Cordova	2016	USA	• Ecodevelopmental theory	Informed analysis and testing of the parent-adolescent family functioning discrepancy hypotheses
Cordova	2020	USA	• Empowerment theory	Informed intervention
			• Ecodevelopmental theory	
Coyle	2019	USA	• Positive youth development framework	Informed intervention and data analysis
			• Social cognitive theory	
DeAtley	2020	South Africa	• Bronfenbrenner’s ecological systems theory	Guided the study and data analysis
Eisenberg	2013	USA	• Social ecological frameworks	Guided study and data analysis
Folayan	2016	Nigeria	• Lazarus and Folkman’s conceptual framework of stress and coping	Informed data analysis
Halkitis (1)	2013	USA	• Fundamental causes theory	Informed data analysis
			• Syndemic theory	
Halkitis (2)	2013	USA	• Singer’s syndemic theory	Informed data analysis
Huebner	2014	USA	• Diaz’s model of social oppression	Tested the model
James	2018	USA	• Social ecological model	Guided the study
Johns	2010	USA	• Social disorganization theory (SDT)	SDT informed hypothesis 2, TGP used to interpret a finding
			• Theory of gender and power (TGP)	
Karamagi	2018	Uganda	• Quality improvement for behavior change model (QBC)	Tested the model’s effectiveness on outcome
Li	2019	USA	• Szapocznik and Coatsworth’s ecodevelopmental theory	Guided the study and data analysis
Logie	2017	Jamaica	• Baral’s social ecological model	Guided the study and data analysis
Mathur	2020	Kenya, Malawi, Zambia	• Proximate determinants theoretical framework	Informed analysis
Maticka-Tyndale	2010	Kenya	• Information motivation behavioral skills model	Informed analysis
			• Campbell’s identification of community influence on HIV risk reduction model	
Miller	2018	USA	• Bernard et al.’s conceptual work on community opportunity structures	Informed analysis
			• Hatzenbuehler et al.’s conceptual work on structural stigma	
Mmari	2013	Uganda	• Risk and protective factor framework	Informed analysis
			• Ecological model	
Mustanski	2019	USA	• National Institute of minority health and health disparities multilevel research framework	Informed analysis
Moodley	2017	South Africa	• Secularization theory	Informed analysis
Nakazwe	2019	Zambia	• Proximate determinants framework	Informed analysis
Njoroge	2010	Kenya	• McLeroy’s social ecological model	Provided multilevel context for the study and for interpretation of findings
Pilgrim	2015	Uganda	• Bronfenbrenner’s ecological system theory	Informed analysis
Placek	2019	India	• McLeroy’s socioecological model	Informed parts of analysis
			• Maternal fetal protection model	
Prado	2010	USA	• Ecodevelopmental theory	Theory tested
Prado	2011	USA	• Ecodevelopmental theory	Guided intervention
Robertson	2010	Multiple countries in SSA	• Expanded Boerma and Weir’s proximate determinants framework	Informed hypothesis development and analysis
Ruisenor-Escudero	2017	Togo	• Modified social ecological model	Informed study conceptualization and analysis
Salud	2014	USA	• AIDS risk reduction model	Informed conceptual framework for the study
			• Acculturation	
			• Theory of gender and power	
Schwandt	2013	Botswana, Malawi, and Mozambique	• Social ecological framework and ideation	Informed intervention and implementation
Ssewamala	2012	Uganda	• Asset theory	Guided study
			• Resilience theory	
Stock	2013	USA	• Prototype/willingness model	Informed analysis
Tenkorang	2014	Kenya	• Information motivation behavioral skills model	Informed analysis
			• Campbell’s community characteristics framework	
Tomita	2017	South Africa	• Social disorganization theory	Guided study and analysis
Tozan	2019	Uganda	• Asset theory	Informed intervention
Tyler	2016	Zambia	• Bronfenbrenner’s ecological framework	Guided study and analysis
Underwood	2015	Multiple countries in SSA	• Theory of economic and social organization	Informed analysis
Waldrop-Valverde	2013	USA	• Socio-ecological model	Informed analysis
Ward-Peterson	2018	Malawi	• Conceptual framework adapted from work by Barnett and Whiteside (2006) and [5]	Guided study and analysis

**Table 2:** List of HIV prevention qualitative and multi-methods articles identified

	Year	Location(s)	Name of multi-level theory/framework	How multi-level theory/framework
HIV prevention (Qualitive n = 21)				
Bird	2017	USA	• Theory of emerging adulthood	Data used to create the Emergent Conceptual Model, named theories provide rationale and used in the interpretation of the new model
			• Developmental approaches to family life-cycle	
			• Family system theory	
			• Structural family therapy	
			• Emergent conceptual model developed by the research team	
Burch	2018	Malaysia	• Modified social-ecological model based on Mustanaski et al. (2011)	Guided study
Casale	2011	South Africa	• Critical social science approach	To develop an interview guide for focus group discussions and data analysis
			• Conceptual framework developed by the research team	
Conn	2013	Uganda	• Framework of gender empowerment and positive sexuality	Framed the narrative analysis; applied to HIV prevention paradigms
Darlington	2012	Jamaica	• Socio-ecological model	Guided focus group discussions; organized themes that emerged from the data
Dyson	2018	USA	• Socio-ecological model	Informed data analysis
Enah	2014	USA	• Model of adolescent sexual risk behaviors	Informed semi-structured interviews and data analysis
			• Elaboration likelihood model	
Harper	2014	Kenya	• Bronfenbrenner’s bioecological systems theory	Informed development of focus group guide and analysis
Hudson	2012	USA	• Comprehensive health seeking and coping paradigm	Guided data interpretation
Hutchinson	2012	Jamaica	• Theory of planned behavior	Informed semi-structure interview guides
			• Parental expansion of theory of planned behavior	
Katz	2013	Uganda	• Explanatory framework of adolescent sexual decision-making	Data used to create the Explanatory Framework
Khan	2018	India	• Structural violence	Informed interpretation of data
			• Moral pragmatics	
			• Foucault state power and discourse	
Kubicek	2015	USA	• Resource theory	Informed framing of research question
Logie	2018	Jamaica	• Syndemics theoretical framework	Guided the study and data analysis
Lyons	2013	USA	• Syndemic theory	Informed research questions
Newman	2013	Thailand	• Socio-ecological models based on Bronfenbrenner’s ecological systems theory	Informed semi-structured interview guide; guided conceptual map and presentation of results
Nwokocha	2015	Nigeria	• Conceptual framework based on structural functionalism, rational choice, and differential association theories	Guided the study
Rahangdale	2010	India	• Modified Steward’s framework on stigma	Informed data analysis and interpretation of results
Richardson	2013	USA	• Anderson’s code of the street	Informed focus group discussion guide and analysis
Stevens	2013	USA	• Integrative model of behavior change	Used to develop the focus group script and Informed analysis
			• Ecological systems theory	
Underwood	2011	Botswana, Malawi, and Mozambique	• Stokol’s social ecological perspective	Informed the analysis
			• Social ecology	
HIV prevention (Multi-methods—quantitative and qualitative n = 2)				
Arrington-Sanders	2016	USA	• Bronfenbrenner’s ecological systems theory	Informed analysis
Cordova	2019	USA	• Empowerment theory	Informed intervention
			• Ecodevelopmental theory	

**Table 3:** List of HIV ‘care and treatment’ and ‘prevention and care and treatment’ articles identified

	Year	Location(s)	Name of multi-level theory/framework	How multi-level theory/framework was used
HIV treatment and care (Quantitative n = 5)				
Jeffries	2017	USA	• Social ecological theory	Informed study analysis
Mutumba	2017	Uganda	• Transactional model of stress and coping	Informed multilevel factors
Naar-King	2013	USA	• Socio ecological model	Hypothesized the association among multilevel factors and non-adherence; assessed analysis
Pantelic	2017	South Africa	• Hypothesized risk pathways from HIV-related disability to internalized HIV stigma	Informed study hypothesis
Nestadt	2019	Thailand	• Modified social action theory	Informed intervention
HIV treatment and care (Qualitative n = 9)				
Ashaba	2019	Uganda	• Conceptual model	Informed the relationship of the study variables
Crowley	2019	South Africa	• Self-management conceptual framework	Guided the study
			• Individual and family self-management theory	
			• Bronfenbrenner’s ecological systems theory	
Galea	2018	Peru	• Social ecological systems theory	To guide analysis and conceptualization of the data
Harper	2019	Kenya	• Disability-stress-coping model	To guide inquiry and analysis
Mutumba	2019	Uganda	• Self-management of chronic diseases framework	Informed multilevel factors
Rutakumwa	2015	Uganda	• Family systems circular causality	Informed interpretation of study findings; guided study implications and future research
Skovdal	2012	Kenya	• Peer social capital framework	Informed study methodology
Wolf	2019	Kenya	• Socio-ecological model	Informed study
Wong	2017	China	• Conceptual model of sexual health disclosure	Guided semi-structured interviews; informed results
HIV prevention and care and treatment (Mixed methods n = 2)				
McKay	2014	USA	• Social action theory	Informed the “CHAMP+” intervention components
Vu	2017	Uganda	• Human rights framework	Guided the “Link Up” intervention
HIV affected youth (Quantitative n = 2)				
Li	2019	China	• Social action theoretical framework	Informed the intervention
Li	2017	China	• Socioecological theories of child development	Informed the intervention
			• Psychological resilience theories	

### Multi-level Approaches

Altogether, the 88 included articles presented a total of 72 different multi-level theories, with about a quarter of the published manuscripts presenting multiple theories. Specifically, 33 (38%) utilized socio-ecological models (SEMs) or the eco-developmental theory. These multi-level approaches often described the components of Bronfenbrenner’s (i.e., macrosystem, exosystem, mesosystem, microsystem) [109] or McLeroy’s (intrapersonal, interpersonal, organizational, community, and public policy) SEMs [110, 111]. Other articles included sociological and structural theories such as the Theory of Gender and Power and Social Disorganization, as well as adolescent-specific theories such as the Theory of Emerging Adulthood. Other examples of theories this review found include Foucault State Power and Discourse, Family System Theory, and the Disability-Stress-Coping Model. Most authors described the approach as informing their data analysis, and 12 out of the 88 articles described the approach as informing or guiding an intervention.

## Discussion

We found 88 articles published between 2010 and May 2020 that fulfilled the criteria for this scoping review, suggesting an opportunity for increased use of multi-level theories and frameworks among researchers in the field of AYA HIV prevention and care and treatment. Most of these articles also focused on AYA HIV prevention, with fewer addressing AYA care and treatment. Most of the included care and treatment literature was published in 2017 and later. This overall lag in HIV care and treatment research may be in part due to the initial focus on advancing treatment options and availability. As treatment has become more widely available, efforts have turned to the behavioral and multi-level aspects of supporting AYAs’ engagement with the care continuum, as reflected in some recent National Institutes for Health requests for applications [112, 113].

This scoping review also highlights a lack of AYA intervention focused research that utilized a named multi-level theory or framework. The view that conducting multi-level interventions is challenging due to its complexity and expense is summarized by Kaufman et al.: “multi-level approaches…are in many ways at odds with contemporary HIV-related policy, which often favors brief, replicable, and easily disseminated interventions” (p. S251) [7]. Such challenges may be amplified when working with AYA whose continued development may result in changing HIV risks and resiliencies across the various levels of a multi-level approach. For example, identity development, puberty, cognitive growth, and age may all lead to greater AYA risk (e.g., alcohol use) as well as resiliencies and protection (e.g., access to clinics/understanding of information). However, given the evolving nature of adolescence, it is critical that we use multi-level theories and frameworks to improve AYA HIV-related health outcomes. Such approaches can be achieved by designing and adapting interventions at selected levels that allow for and are responsive to AYA developmental needs. For example, Denison et al. pilot-tested the “Family Connections” family-based intervention among AYAs, ages 15 to 19 years, living with HIV in Zambia. Based on positive youth development, Family Connections moved beyond individual level factors to engage the family caregivers (interpersonal level) and health care providers (environmental level) [114]. To expand on this pilot study, the team is now conducting a National Institute for Mental Health—funded R01 to examine both the impact of Family Connections on youth achieving an undetectable viral load, and if developmental differences among participants (e.g., cognitive functioning, emotional regulation and impulse control) moderate any impact found. Studies that combine multi-level theory and incorporate developmental factors into intervention testing illustrate how we may strengthen our AYA HIV research to engage and address the needs of AYAs.

Our scoping review findings also highlight the ways researchers creatively drew upon different theories and frameworks to examine multi-level factors within their respective studies. This practice of drawing upon different theoretical perspectives is an important contribution and supports the recommendation of Kaufman et al. to utilize existing theories at various levels until a new theory is needed [7]. In the scoping review, we found that authors sometimes combined individual and/or structural theories with SEMs. This process can help translate SEMs, which tend to be broadly applied, to specific populations and factors, explicitly detailing proposed hypotheses of how change occurs. We recommend researchers continue to combine theories to clearly link and measure multi-level variables and their interactive effects on behavior change.

### Adapted AYA HIV Multi-level Framework

**Fig. 2:**
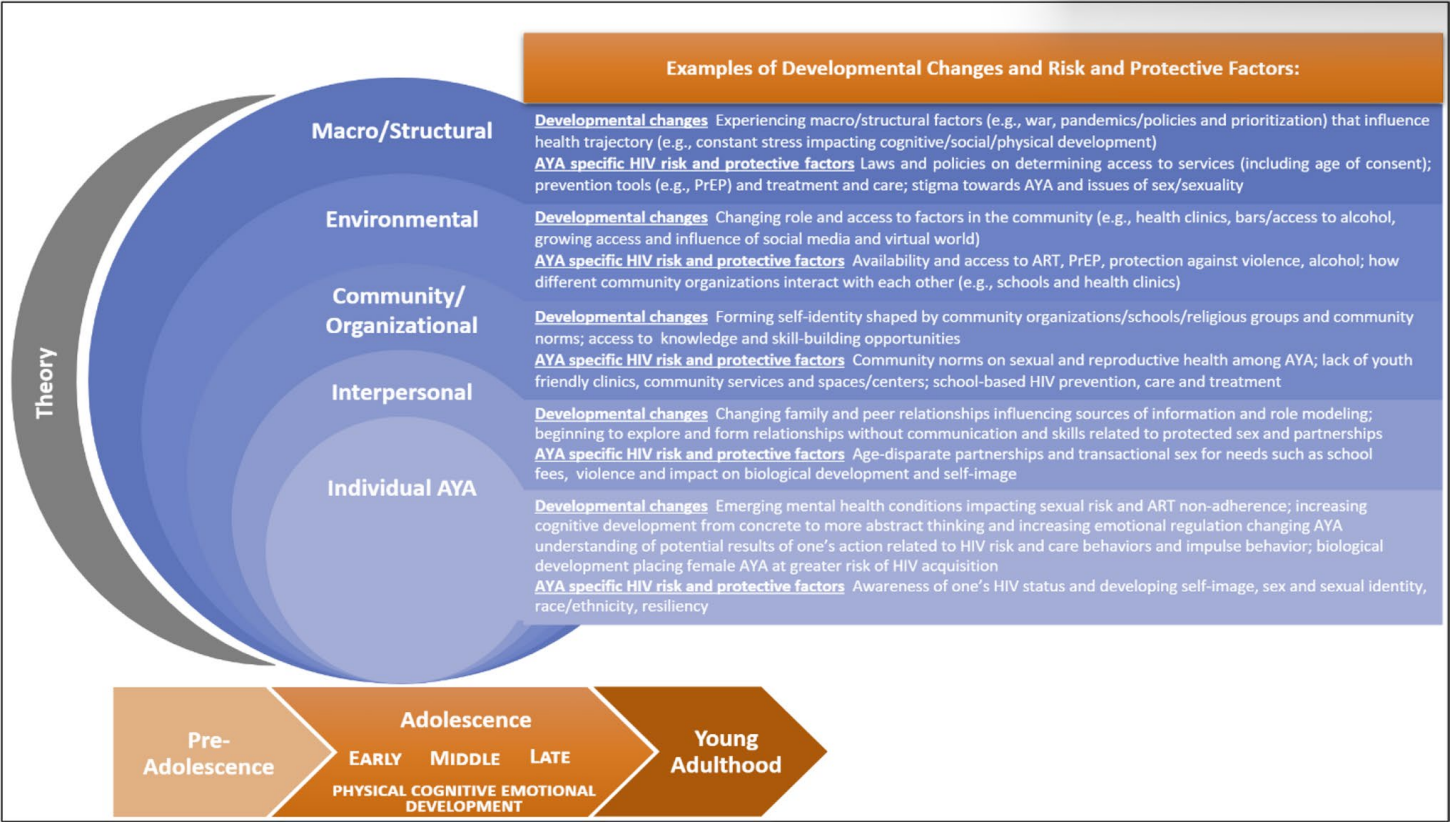
A tool to support multi-level approaches in AYA HIV research Adapted from the WHO Adolescent Health Ecological Model (16)

Given the importance of multi-level theories and frameworks for advancing AYA HIV research, and the lessons learned from this scoping review, the interdisciplinary JHU CFAR AYA SWG adapted the WHO’s Adolescent Health Ecological Model to HIV specifically. In this adapted framework we explicitly emphasize the dynamic and changing nature of adolescence within the context of HIV (Figure 2). The arrow across the bottom underscores the broad developmental stages of adolescence and young adulthood within a life course perspective. To make the framework more parsimonious and accessible, we also collapsed four of the original seven levels. We combined community and organizational levels into one level to group the social norms (e.g., values, networks) and institutions (e.g., schools) that may exist within an AYA’s broader community. We also combined the macro/structural levels that encompass super structural (e.g., war, racism) and structural (e.g., policies, laws) factors. Within each level, we highlight AYA developmental changes and provide examples of the HIV-specific risk and protective factors that may be at play. In the adapted framework, we also remind researchers to use theory, as shown in the grey moon-shaped sliver in the figure, to guide intervention design, measures, and analyses, and to explicitly state how factors across levels are hypothesized to interact and impact AYA HIV outcomes. Overall, the goal of this adapted framework is to provide interdisciplinary teams of AYA HIV researchers with a tool for conceptualizing the developmental changes and the corresponding HIV risk and protective factors they could consider in their interventions and to state the theoretical relationship among these variables guiding their analyses across levels.

### Limitations

There are limitations to the scoping review. First, only published articles in English that had the search terms, such as theoretical framework, in the title/abstract or as a MeSH term, were included. This process may have missed articles that used multi-level theories or frameworks that did not include the terms we used to search. This process also excluded grey literature and any published article in a language other than English, potentially resulting in publication and language biases respectively. Finally, we did not assess the use of multi-level analytic models, interventions, or study designs in the absence of a named theory, or the quality of the research in the included articles.

## Conclusions

This scoping review highlights a paucity of published articles that utilized a named multi-level theory or framework, particularly in HIV care and treatment. The scoping review also found that researchers who have used multi-level theories or frameworks have taken creative approaches to integrate theories and/or have relied on socio-ecological models. Use of multi-level approaches by AYA HIV researchers is greatly needed, and we offer an adapted model to facilitate these efforts.

## Data Availability

The authors confirm that the data supporting the findings of this study are available within the article.

## Appendix

See Table 4

**Table 4:** Search terms

	Concept #1 HIV	Concept #2 Adolescent	Concept #3 Conceptual models
PubMed	HIV[mesh] OR HIV[tiab] OR AIDS Virus*[tiab]	Adolescent[Mesh] OR Young Adult[Mesh] OR Adolescen*[tiab] OR Teen*[tiab] OR Youth*[tiab] OR young adult*[tiab] OR student*[tiab]	Models, theoretical[mesh] OR theoretical model*[tiab] OR conceptual model*[tiab] OR theoretical framework*[tiab] OR conceptual framework*[tiab] OR social-ecological model*[tiab] OR socio-ecological model*[tiab] OR multi-level[tiab] OR multilevel[tiab] Filters: from 2010/1/1 - 2020/5/31
Embase	'Human immunodeficiency virus'/exp OR (‘HIV’ OR ‘AIDS virus*’):ab,ti,kw	‘Adolescent’/exp OR ‘Young Adult’/exp OR (‘Adolescen*’ OR ‘Teen*’ OR ‘Teen’ OR ‘Youth*’ OR ‘young adult*’ OR ‘student*’):ab,ti,kw	‘Theoretical model’/exp OR ‘conceptual framework’/exp ’OR 'multilevel analysis'/exp OR 'social ecological model'/exp OR (‘theoretical model*’ OR ‘conceptual model*’ OR ‘theoretical framework*’ OR ‘conceptual framework*’ OR ‘social ecological model*’ OR ‘socio ecological model* OR multilevel OR 'multi level'):ab,ti,kw
CINHAL	(MM "Human Immunodeficiency Virus") OR “HIV” OR “AIDS virus*”	MM "Adolescent" OR MM "Young Adult" OR "Adolescen*” OR "Teen*" OR "Youth*" OR "young adult*" OR “student*”	(MM "Models, Theoretical") OR (MM "Conceptual Framework") OR "theoretical model*" OR "conceptual model*" OR "theoretical framework*" OR "conceptual framework*" OR “social ecological model*” OR “socio ecological model*” OR multilevel OR multi-level
PsycINFO	DE "HIV" OR DE "AIDS" OR “HIV” OR “AIDS virus*”	“adolescen*” OR “young adult*” OR “teen*” OR “youth*” OR “young adult*”	“theoretical model*” OR “conceptual model*” OR “theoretical framework*” OR “conceptual framework*” OR “social ecological model*” OR “socio ecological model” OR multilevel OR multi-level
Sociologic Abstracts	ab,ti,su(HIV) OR ab,ti,su(AIDS virus)	ab,ti,su(Adolescen*) OR ab,ti,su(Teen*) OR ab,ti,su(Youth*) OR ab,ti,su(young adult*) OR ab,ti,su(student*)	ab,ti,su(theoretical model*) OR ab,ti,su(conceptual model*) OR ab,ti,su (theoretical framework*) OR ab,ti,su(conceptual framework*) OR ab,ti,su(social ecological model*) OR ab,ti,su(socio ecological model*) OR ab,ti,su(multilevel) OR ab,ti,su(multi-level)

**MM *major concept, *DE* subject {exact} (explode), *MH* explode
